# Older cancer patients and COVID‐19 outbreak: Practical considerations and recommendations

**DOI:** 10.1002/cam4.3517

**Published:** 2020-11-21

**Authors:** Antonella Brunello, Antonella Galiano, Silvia Finotto, Silvio Monfardini, Giuseppe Colloca, Lodovico Balducci, Vittorina Zagonel

**Affiliations:** ^1^ Oncology 1 Unit Department of Oncology Istituto Oncologico Veneto IOV ‐ IRCCS Padova Italy; ^2^ Istituto Palazzolo Fondazione Don Gnocchi Milano Italy; ^3^ Dipartimento di Diagnostica per Immagini Radioterapia Oncologica ed Ematologia Istituto di Radiologia Fondazione Policlinico A. Gemelli IRCCS ‐ Università Cattolica Sacro Cuore Roma Italy; ^4^ Moffitt Cancer Center University of South Florida College of Medicine Tampa Florida USA

**Keywords:** cancer, chemotherapy, COVID‐19, elderly, guidelines, management, treatment

## Abstract

Since the COVID‐19 outbreak started, it has been affecting mainly older individuals. Among the most vulnerable older individuals are those with cancer. Many published guidelines and consensus papers deal with prioritizing cancer care. Given the lack of high‐quality evidence for management of cancer in older patients also in normal times, it is even more stringent to provide some resources on how to avoid both undertreatment and overtreatment in this population, who as of now is twice challenged to death, due to both a greater risk of getting infected with COVID‐19 as well as from cancer not adequately addressed and treated. We hereby discuss some general recommendations (implement triage procedures; perform geriatric assessment; carefully assess comorbidity; promote early integration of palliative care in oncology; acknowledge the role of caregivers; maintain active take in charge to avoid feeling of abandonment; mandate seasonal flu vaccination) and discuss practical suggestions for specific disease settings (early‐stage and advanced‐stage disease for solid tumors, and hematological malignancies). The manuscript provides resources on how to avoid both undertreatment and overtreatment in older patients with cancer, who as of now is twice challenged to death, due to both a greater risk of getting infected with COVID‐19 as well as from cancer not adequately addressed and treated.

## 1 INTRODUCTION

As of today (31st August 2020) more than 25,000,000 subjects have been recognized worldwide to be infected with SARS‐CoV‐2 (Severe Acute Respiratory Syndrome Coronavirus 2), with almost 900,000 deaths. Italy, Spain, and France in Europe have been the first affected countries, along with the United Kingdom and, outside Europe, United States of America as well as Brazil have the highest number of deaths as well as of infected subjects.^1^

The impact of respiratory virus infections on morbidity and mortality in patients with cancer is widely recognized, with a risk of being hospitalized which is fourfold higher compared to age‐matched subjects.^2^

In this epidemiological scenario, older persons with cancer are at particularly high risk of adverse outcomes,^3^, ^4^ because of their actual risk of getting the disease, as well as for a higher likelihood of being denied proper and timely cancer treatment in order to protect them from COVID‐19 exposure.^5^ As in other disaster medicine scenarios, ethical dilemmas are posed which are not easy to resolve and may be matter of debate.^6^ Patient and staff safety are of utmost importance. Yet, through the turmoil created by COVID‐19, we must keep in mind that about 10 million people will die from cancer this year, and about half will be 70 years or older.^7^ Predicting when the outbreak will end, even at a local level, remains a challenge. Therefore, health‐care systems, along with individual providers, must work within institutional policies aiming at reducing infections, ensuring sufficient resources, and providing safety for patients and health‐care staff, in order to deliver optimal cancer treatment.

## 2 CHALLENGES IN THE CARE OF OLDER CANCER PATIENTS DURING COVID‐19 PANDEMICS

Managing care of older patients with cancer is an issue of high controversy, given the relative paucity of evidence to guide decisions even in normal times. In a context in which the global population is aging and cancer incidence increases in the older cohorts, the difficulties in deciding the most appropriate treatment for older cancer patients have been all of a sudden replaced by the void and anxiety related to the objective difficulty of making choices, given the major challenges posed by the outbreak of COVID‐19 epidemics, with risks of undertreatment as well as overtreatment never been so high as it is now.

This paper expresses the position of the authors who are oncologists, hematologists, and geriatricians, to recall and emphasize the general recommendations that commonly apply to management of older patients with cancer, as well as to provide specific guidance and suggestions to safely handle care of older cancer patients during the COVID‐19 epidemics.

## 3 GENERAL RECOMMENDATIONS

### 3.1 Implement triage procedures

Early diagnose of COVID‐19 infection in patients with cancer may lead to earlier take in charge and better outcomes, and eventually to better care for cancer itself. Therefore, timely recognition of symptoms with accurate initial medical history and physical examination should be recommended. Though as of now no specific therapy has demonstrated clear effectiveness, an early take in charge would mean strict monitoring and avoiding getting proper treatment in late phase of disease. A note of caution must be made for all patients under steroid treatment, due to oncological reasons or to comorbidity, which could lead to underestimation of dyspnea and/or fever.

Patients with lung cancer, and to some extent patients with lung metastases from other neoplasms, may have compromised lung function with associated symptoms such as polypnea, dyspnea, or cough, which make them at higher risk of severe forms of COVID‐19 infection. Early Chinese data in fact show that lung cancer was the most prevalent (28%) type of malignancy in a cohort of COVID‐19‐infected cancer patients,^8^ with subsequent evidence pointing at increased risk of negative outcomes in patients with thoracic malignancies.^9^

Based on these data, older patients with lung cancer or other thoracic malignancies should be carefully studied with regard to COVID‐19 symptoms, and assessed with CGA and physical performance tests in order to have all best possible elements for decision‐making.

### 3.2 Perform geriatric assessment

COVID‐19‐related mortality is almost a prerogative of older and frail subjects. Older patients with cancer are at very high risk of dying if they are infected, with COVID‐19 becoming a sort of frailty stress test.

A growing body of evidence has shown multiple benefits from a comprehensive geriatric assessment (CGA), and its usefulness in oncology has been known for more than 30 years.^10^ Geriatric assessment is time‐consuming, yet, it has been stressed several times that CGA costs compared to other standard assessment commonly used for decision‐making in cancer patients are much lower, while carrying expected larger benefits.^11^

In “normal times” the output of a CGA is often overlooked, since cancer‐related parameters are easier to use and friendlier to oncologists compared to patient‐related factors. Yet, in times of epidemics, patient‐related factors are increasingly being considered by several scientific societies as priorities, with some scientific societies addressing the problem of older cancer patients and in some cases advising not to see patients older than 70 years old in the clinics.^12^, ^13^, ^14^

CGA domains can provide useful scores to help defining prognosis,^15^, ^16^ which is a fundamental step to take in decision‐making for cancer‐directed treatment, particularly during the pandemic.

CGA‐based scores are also available to help predicting toxicity from chemotherapy, the ones more extensively applied so far being the Cancer and Aging Research Group (CARG) score and the Chemotherapy Risk Assessment Scale for High‐Age Patients (CRASH) score.^17^, ^18^

Given this background, our strong recommendation is to assess older cancer patients with CGA and an accurate measure of physical performance, which will provide better prognostic and predictive ability compared to “simple” use of clinical judgment and performance status.

### 3.3 Carefully assess comorbidity

Comorbidity is known to impact cancer treatment, increasing risk of toxicity from chemotherapy as well as from target agents.^19^, ^20^

Also, comorbidity may be worsened by cancer‐directed treatment, and this is quite a relevant issue if we consider the increased risk of decompensating diabetes with steroids or worsening of hypertension due to anti‐VEGF targeted agents.

It is not yet known why some comorbidities seem to put COVID‐19 patients at greater risk. Indeed, diabetes and cardiovascular disease are among the major risk factors for severe course of COVID‐19 in infected subjects,^21^, ^22^ with Italian data showing that death from COVID‐19 occurs mainly in subjects aged 70 years and older, with less than 1% patients having no associated disease.^23^

A recent meta‐analysis of studies that summarized the prevalence of cardiovascular metabolic diseases in COVID‐19 and compared the incidences of the comorbidities in patients with severe and non‐severe course of disease showed that hypertension, cardiac and cerebrovascular diseases, and diabetes were from twofold to threefold higher in severe cases compared to non‐severe ones.^24^

Moreover, patients with diabetes are at risk of infections, especially influenza and pneumonia, and this risk can be reduced by optimal glycemic control. Diabetes was recognized as an important risk factor for mortality in patients infected with H1N1 influenza virus and Severe Acute Respiratory Syndrome (SARS) and Middle East Respiratory Syndrome‐related coronavirus (MERSCoV). Therefore, for diabetic patients who are in need of active cancer treatment, lowering risk of respiratory infections through pneumococcal and annual influenza vaccinations should be recommended.^25^

In light of these considerations, given the high prevalence of multimorbidity in the older cancer patients’ population, careful assessment of presence of associated disease, their treatment and control status is mandatory along with revision and reconciliation of poly‐pharmacotherapy.

### 3.4 Promote early integration of palliative care

With evidence accumulating on the role of early palliative care/simultaneous care in oncology, all efforts should be made to grant early access to palliative care for patients with advanced‐stage disease receiving disease‐modifying treatment as well as to provide adequate symptom control. This applies both to patients with solid cancers^26^ and with hematologic malignancies.^27^

In the home care setting, great attention must be paid to avoiding the risk of patients getting infected, which can lead to severe and life threatening forms of COVID‐19, as well as to avoid the risk of the health‐care personnel of being infected. Specific protocols to ensure safe take‐in‐charge for home services are being developed.^28^

In view of the high risk of mortality and suffering related to the clinical evolution of respiratory insufficiency that mostly afflict patients with COVID‐19, early palliative care is even more important when active cancer‐directed treatment is avoided and when COVID‐19 infection occurs in frail older cancer patients.

### 3.5 Acknowledgement the role of caregivers

It is of utmost importance to instruct caregivers to diligently observe actual government and WHO instructions in order to avoid unnecessary exposition of older cancer patients to COVID‐19 risks. In order to provide better assistance, help relieving the burden of caregivers, and improve their psychological symptoms, early integration of palliative care should be pursued for older patients with advanced‐stage cancer and/or symptomatic disease.^29^

In the management of older cancer patients during the epidemics, the caregiver reveals a role of fundamental importance. There is not only the need to "accompany" the patient with treatments, but also to avoid that episodes of infection can occur and, on the contrary, to identify the first symptoms of a possible infection.

The evaluation of caregivers’ stress becomes crucial, given their primary role of allowing treatment to be safely delivered and preventing patients from becoming infected.^30^

### 3.6 Maintain active take in charge to avoid feeling of abandonment

Oncological follow‐up is just as important as active treatment phase and, in a time in which ambulatory visits are being canceled or reduced, alternatives routes to classical outpatient visits are a good way to overcome the distress of the patients and their families and to avoid the feeling of abandonment. In a society where the diagnosis of cancer has a significant impact, the loneliness, abandonment, or anguish of not being able to be treated must be absolutely avoided.

1. In this light, proper activation of web‐based resources to communicate with oncologists and tele‐consultations may help provide continuity of oncological take in charge.
2. Also, given the high burden of distress the pandemics may exacerbate, psychological tele‐consultations should be provided.

### 3.7 Mandate seasonal influenza vaccination

Many Countries, Italy included, provide free vaccination for older subjects (>65 years old) through general practitioners.

Oncology units should make it mandatory, in the absence of proved contraindications, to have all cancer patients older than 65 years get seasonal flu vaccine, since even in the absence of clear data on cross‐protection, there would be one less competitive factor for respiratory disease.

## 4 PRACTICAL SUGGESTION

**FIGURE 1:**
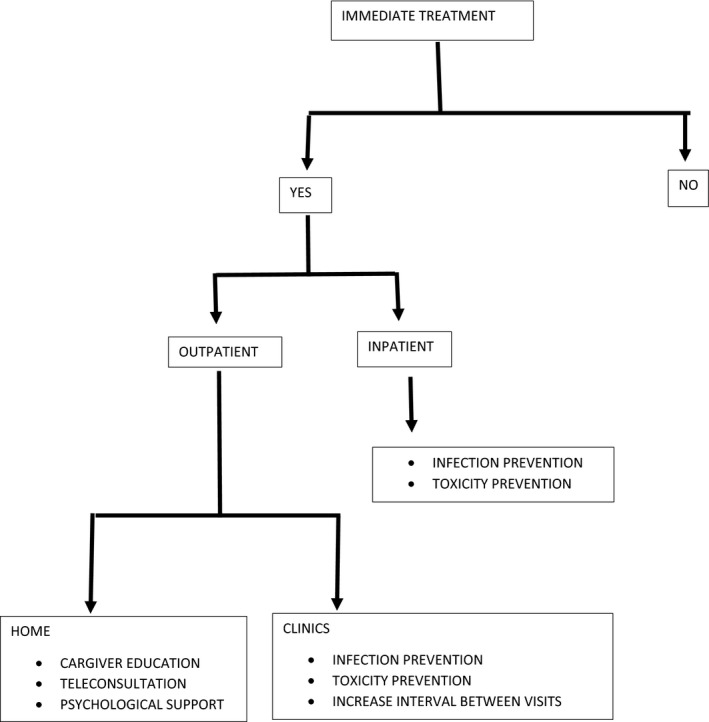
Management algorithm for older patient with cancer

Bearing in mind that older patients will be those suffering more from strict policies of prioritization of health interventions for patients with COVID‐19, in order to avoid arbitrary discrimination based on age and to allow better allocation of treatment we propose a treatment algorithm for older patients with cancer during the COVID‐19 pandemics (Figure 1), in accordance with precautionary principles.^31^

A first pivotal question regards the necessity of immediate treatment: oncological judgment on biological behavior of neoplasm is vital to consider whether postponing treatment would be adequate and appropriate. As examples of scenarios in which treatment may be deferred are patients with small metastases with slow growth, indolent forms of chronic lymphocytic leukemia, or asymptomatic lymphoma.

On the contrary, if immediate systemic treatment is indicated, then, the next decisional node would be whether treatment is delivered in an outpatient setting or if it requires hospital admission. Unless the goal of treatment is curative, outpatient setting should be always preferred, given the high risk of nosocomial infections.

For the outpatient setting, a further decision node involves identifying necessity of treating patient in the clinics with intravenous drugs, or considering the possibility of delivering treatment orally at home.

All the branches of the algorithm with in‐hospital treatment requirement, be it as inpatient or outpatient, require a careful assessment of factors for risk minimization.

Every effort should be put in preventing infection and preventing toxicity.

### 4.1 How can infection be prevented?

Since many COVID‐19 infections are nosocomial, aggressive measures should be undertaken to reduce frequency of hospital visits of patients. For patients who require treatment, proper isolation protocols must be put in place to mitigate the risk of SARS‐CoV‐2 infection.

COVID‐19 cases requiring inpatient care should be transferred to a specialized facility as soon as possible, in order to avoid cross‐transmission.

Limiting access to hospital includes policies of longer intervals between visits. For solid malignancies such as breast cancer and prostate cancer most patients would receive endocrine treatment, with benefits of adding targeted agents (i.e., cdk 4/6 inhibitors) to be accurately weighted against the risks deriving from increased side effects. Many patients on such “chronic” treatment could benefit from drugs being home‐dispatched.

For solid tumors requiring cytotoxic chemotherapy, options for treatment de‐escalation^32^, ^33^ or therapeutic breaks^34^ should be pursued.

Patients requiring treatment with bone resorption inhibitors may be switched to every 3 months schedule instead of the monthly schedule.^35^

Anti‐PD‐(L)1 cycles may be modified/delayed to reduce clinical visits; for instance, using 4‐ or 6‐weekly instead of 2‐ or 3‐weekly schedules when appropriate and if allowed by national regulatory agencies. Also, evidence has become available for therapeutic breaks in patients treated with immune checkpoint inhibitors with controlled disease, after at least 1 year.^36^

In this scenario, oral therapies limit the number of ambulatory visits thus reducing the nosocomial risk which is mostly related to hospital access, and can frequently be proposed as good alternatives to intravenous treatments, with evidence suggesting that low‐dose or metronomic schedules may be as effective as standard scheduling, with lesser side effects.^37^, ^38^

Therefore, whenever available oral therapy should be preferred over intravenous therapy, and telephone and/or web‐based contact should be planned to follow the course of therapy.

Also, whenever possible, biochemical and imaging studies should be postponed, especially for patients in follow‐up or patients with long‐term stable and/or indolent disease.

Privileging oral route will also avoid the necessity of central catheter use and as well reduce risks related to central catheters such as infections and thromboembolism.

Again, and even more important in the setting of oral therapy, the presence of caregiver and early activation of palliative care home services are imperative.

Of note, education of patients and caregivers is of vital importance both for treatment administered in the clinics as well as for patients at home, since contacts with caregiver could be a source of infection too. Primary prevention of infection must be enforced, and patients and caregiver instructed to wear masks, wash hands correctly and regularly, and avoid any direct contact which is not necessary.

### 4.2 How can toxicity be prevented?

In general, when considering classical cytotoxic chemotherapy, expected toxicities are very well known. Hematological toxicity is easier to predict, and is highly related to regimen and schedule of treatment. Among the most worrisome toxicities in times of an epidemic is leukopenia, which is dependent on the cytotoxic regimen as well as on patient‐related factors.

Recently, a risk score (“FENCE” score) for febrile neutropenia after chemotherapy has been made available, both at the first cycle of chemotherapy^39^ and for cycles 2‐6 in patients with solid cancers.^40^

Non‐hematological toxicity is not‐so‐easy to predict, yet, the CARG score and the CRASH score can be highly helpful.^17^, ^18^

Thus, one major practical recommendation for all older patients being considered for cytotoxic chemotherapy is to use these, or other disease‐specific CGA‐based prognostic scores, to estimate risk of toxicity from chemotherapy, and refrain from using cytotoxic chemotherapy if risk of toxicity is higher than 40% and not preventable, that is, by using prophylactic Granulocyte Colony‐Stimulating Factor (G‐CSF) and/or reducing dose/intervals in palliative setting.

Besides that, if cytotoxic chemotherapy is highly indicated, preference should be given to regimens with lower predicted toxicity whenever possible.

Targeted agents are being used more and more, often instead of antiblastic chemotherapy, with usually a better safety profile which, in general, rarely poses a threat to life.^41^ However, the use of such drugs in older patients raises doubts about therapeutic adherence, given that even a grade 2 toxicity may not be bearable for long time periods, and the risk of interaction with the usual polypharmacy, with possible consequences on quality of life.^41^, ^42^

The heterogeneous mechanisms of action of these agents (i.e., epidermal growth factor receptor, vascular endothelial growth factor receptor, and proteasome inhibitors), combined with additional factors such as decreased creatinine clearance, performance status, age, and comorbidities make prediction of treatment toxicity difficult, though predictive nomograms have been developed.^43^, ^44^

As for immune checkpoint inhibitors, apart from known baseline conditions which could carry a high risk of toxicity such as preexisting autoimmune disease, there is no evidence‐based predictive factor for higher toxicity in older patients, yet, the risk of potential immune checkpoint inhibitor‐induced pneumonitis needs to be taken into account for differential diagnosis, actively looked for and promptly treated.

Finally, a vital tip for preventing toxicity in older cancer patients is to remove potential inappropriate medication, providing careful revision and reconciliation of polypharmacotherapy.

## 5 RECOMMENDATIONS FOR MANAGEMENT IN SPECIFIC SETTINGS

General as well as specific‐setting recommendations for medical treatment are summarized in Table 1.

**TABLE 1:** Recommendations for management of older patients with cancer during COVID‐19 pandemic.

Setting	Recommendation	Suggestion
General Considerations	Use CGA‐based prognostic scores to estimate risk of toxicity from chemotherapy & prevent toxicity	‐ use supportive measures (i.e., G‐CSF in primary prophylaxis, treatment of underlying anemia, prevention of mucositis); ‐ avoid using cytotoxic chemotherapy if risk of toxicity is higher than 40% and not preventable (i.e., by using prophylactic G‐CSF) ‐ review and reconcile polypharmacy ‐ If cytotoxic chemotherapy is highly indicated, use regimens with lower predicted toxicity if possible
	Prevent infections	‐ Implement triage procedures ‐ Engage with caregivers ‐ Avoid giving treatment as inpatients ‐ Limit patients’ travels (i.e., use oral drugs if possible; dispatch drugs at home if chronic and side effects verified; interact with home care services; switch to longer interval schedules if possible) ‐ Consider watchful waiting/postponing treatment for low volume, biologically indolent tumors; ‐ Consider use of local treatment when possible (i.e., radiation therapy) to avoid / delay systemic treatment ‐ Vaccinate for influenza
Early‐stage disease	Use CGA‐based prognostic tools to identify patients who may derive benefit from (neo)adjuvant chemotherapy	If (neo)adjuvant treatment is indicated use supportive measures to prevent toxicity and infection
Advanced‐stage disease	Use prognostic tools to estimate prognosis of patients with advanced stage disease in decision‐making	Do not start any oncological treatment if patients have less than 3 months life expectancy, unless poor prognosis is mainly related to cancer and treatment is likely to have major impact on disease course with no severe toxicity expected
		Do not start second or further treatment lines if there was no benefit from prior interventions, and in the absence of possible disease‐modifying new agents with safe toxicity profile
	Assess the goals of treatment and discuss openly with patient	Use single‐agent therapy if goal is prolongation of survival; use combination therapy if goal is tumor shrinkage (for symptoms; for possible conversion to surgery) and if the above “General Considerations” are respected

### 5.1 Systemic therapy

**In the setting of early‐stage disease** for solid tumors we recommend using CGA‐based prognostic tools to help identifying patients who may live long enough to derive benefit from (neo)adjuvant chemotherapy. For patients with early‐stage disease, best prognosticators are those predicting mortality at 5 to 10, with Lee‐Schonberg index being particularly useful in this setting.^45^

Given the evidence demonstrating that adapted and “elderly‐friendly” regimens in older patients are in many cases less effective than standard chemotherapy,^46^, ^47^, ^48^ every effort should be put in place to grant the best possible supportive measures for safe delivery of chemotherapy. In this context, for example, identifying anemia and treating it, and using prophylactic G‐CSF may help preventing both toxicity and dose‐reductions which could impact oncological outcomes.

**In the setting of advanced‐stage disease** oncological treatment (cytotoxic chemotherapy, targeted agents, and immune checkpoint inhibitors) can be considered as a reasonable option in the majority of cases.

Together with the goal of therapy, which is normally palliative in nature, the potential toxicity profile of drugs and the patient's functional status, comorbidity burden, and social support are major points that must be taken into account in the choice of the optimal treatment for older patients with advanced‐stage disease. Targeted therapy and immunotherapy should be preferred over cytotoxic chemotherapy when available.

Cytotoxic treatments should be definitely avoided in patients with poor performance status and more generally no oncological treatment should be started if patients have less than 3 months life expectancy, unless poor prognosis is mainly related to cancer and treatment is likely to have major impact on disease course with no severe toxicity expected.

Despite the lack of high‐level evidence of the utility of performance status to predict outcome from newer regimens, recent data point to the ineffectiveness of even immune checkpoint inhibitors treatment when performance status is poor, particularly in those cases in which the cancer is the leading cause of performance status decline.^49^

Also, to reduce toxicity burden, in the advanced care setting for older cancer patients we should prefer single‐agent therapy, with less frequent scheduling whenever possible, and resort to combination therapy only if goal is tumor shrinkage (i.e., for reducing symptoms; for possible conversion to surgery) and if patient's assessment by CGA indicates fitness for combination therapy. Moreover, when possible, we should consider use of local treatment instead of systemic treatment (i.e., radiation therapy).

One of the major issues in this context is the great difficulty to proper predict survival in patients with advanced‐stage disease. Various prognostic models may aid clinicians in predicting patient survival, and web‐based tools such as www.predictsurvival.com ^50^ can help providing survival prediction based on multiple prognostic scores.

For patients with progressing disease after first line treatment, we can resort to ASCO five key statements to improve care, which remind us not to use cancer‐directed therapy for patients with solid tumor with the following characteristics: low‐performance status (3 or 4), no benefit from prior evidence‐based interventions, not eligible for a clinical trial, and no strong evidence supporting the clinical value of further anticancer treatment.^51^ In this landscape, we should refrain from starting second or further treatment lines if there was no benefit from prior interventions, and in the absence of possible disease‐modifying new agents with safe toxicity profile. On the contrary, as already stressed, timely interaction with home care services and early palliative care should be mandatory.

**In the setting of hematological malignancies**, the same general considerations made for patients with solid tumors hold true, yet, with the caveat that patients with onco‐hematological conditions are at greater risk of infection both for the immunosuppressed status and as a result of treatments. Even if frailty has different implications in different hematological diseases, prognosis should be accurately estimated using CGA‐based assessment tools, which have been showed to be prognostic also in some onco‐hematological setting.^52^, ^53^, ^54^, ^55^

The recommendation to limit patients’ travels to what is strictly needed also holds true for onco‐hematological setting, as well as preferring oral therapies when possible.

A multidisciplinary approach that includes radiotherapists is to be promoted in this moment; indeed radiation therapy could be a valid option to delay chemotherapy or to reduce exposition to systemic therapy, and therefore, to immunosuppression.

For patients treated with chemotherapy with predicted risk of neutropenia >10%, use of primary prophylaxis with G‐CSF is recommended.

During the epidemics, greater attention must be reserved to use of steroids, which play a major role in disease control in hematological patient since in addition to immunosuppressive action, they could cover some early COVID‐19 manifestation such as fever.

With regard to the recently released ASH resources,^12^ some recommendations for specific hematological malignancies treatment can be applied to in older patients (i.e., watchful waiting for indolent disease; prefer oral therapy over i.v. when possible).

### 5.2 Surgery in older patients with cancer during the pandemic

Surgery remains one of the pillars of cancer treatment in older patients as well as in younger ones. Guidelines that impose reduced utilization of cancer surgery can dramatically impact oncological outcomes. Since the COVID‐19 outbreak started, many guidelines for resource allocation have been proposed.^56^, ^57^ As an overarching principle all patients should receive appropriate and timely surgical care, including operative management, based on sound surgical judgment and availability of resources.

Prioritization of treatment should be applied to surgery, as well as to systemic treatment, basing the indication on patients’ symptoms, general health status – assessed through CGA, setting, type of surgery and risk of complications, type of anesthesia, along with availability of hospital resources.

For asymptomatic patients that test negative for COVID‐19, anesthetic and surgical procedures should be conducted under standard operating protocols. For patients who test positive for COVID‐19, surgical procedures should be delayed when possible, both to minimize exposure to health‐care workers and to reduce postsurgical risk. For those patients who test positive for COVID‐19 who need immediate surgery, enhanced operating room management protocols must be in place to reduce viral exposure to health‐care personnel. Indeed, with regard to patients with perioperative SARS‐CoV‐2 infection, an international cohort study has shown postoperative pulmonary complications occur in half of the cases and are associated with high mortality, especially in men aged 70 years and older. For these patients, postponement of nonurgent procedures, or resorting to nonoperative treatment, should be considered.^58^

Omitting surgery could be an option in selected cases when expected benefits are not clear and especially if safer alternative options are available, such as the use of primary endocrine therapy for older patients with early‐stage ER‐positive, HER2‐negative breast cancer^59^, ^60^ or radiation therapy for prostate cancer.^61^

If surgery needs to be postponed, some patients may be candidate for pre‐habilitation, which could help establish a baseline functional level, identify impairments, and provide interventions in order to reduce postoperative morbidity and mortality.^62^ Moreover, in the setting of the COVID‐19 pandemic, pre‐habilitation may counteract the unintended sequelae of physical distancing that may result in decreased fitness arising from increased sedentary behavior, which may in turn lead to increased morbidity and mortality, particularly in vulnerable older patients. Pre‐habilitation has been shown to be feasible also in telehealth programs, therefore, minimizing the risk of COVID‐19 transmission.^63^

### 5.3 Radiation therapy in older patients during the pandemic

Emerging recommendations on multidisciplinary cancer treatment during the COVID‐19 pandemic indicate a shift in radiotherapy indications and a potentially increased demand for radiotherapy,^64^ and several disease‐specific consensus recommendations have been issued.^65^, ^66^

Importantly, in a risk‐mitigation pandemic scenario where radiotherapy resources remain available, efforts should be made to not compromise the prognosis of patients by departing from guideline‐recommended radiotherapy practice. In a severe pandemic scenario characterized by reduced resources, when patients must be triaged important factors for decision‐making include the potential for cure, relative benefit of radiation, life expectancy, and CGA.

Generally, multidisciplinary expert recommendations have been made in the current COVID‐19 pandemics in order to encourage modified treatment strategies, such as increased use of radiotherapy or chemoradiation instead of surgical treatment for defined patient groups with head‐and‐neck cancer, lung cancer, cervix cancer, esophageal cancer, and prostate cancer.

For example, in older patients with early‐stage non‐small cell lung cancer, stereotactic body radiotherapy could be considered as an alternative to lobectomy, in order to reduce potentially prolonged admissions, surgical risks as well as to avoid necessity of monitoring in postsurgical intensive care unit.^67^

In order to minimize travels and hospital access, total treatment time can be reduced by hypofractionation, which has been proven to be safe and effective in multiple randomized trials of various malignancies for both curative and palliative indications.^68^, ^69^ Also, a short course of neoadjuvant radiotherapy could be preferred for a potential cure in older patients with locally advanced rectal carcinoma.^70^ Postponement of radiation therapy up to 5‐6 months can be safely resorted to in some cases, such as patients with early breast cancer treated with chemotherapy and patients with early prostate cancer in case of low‐risk disease, whereas in other cancers, such as head and neck cancer, generally postponing beyond 4‐6 weeks usually is not advisable.^71^, ^72^, ^73^

Radiotherapy can be omitted for older patients with low‐risk breast cancer and in early‐stage Hodgkin's lymphoma.^74^, ^75^

In the palliative setting, single fraction treatment to treat bone pain from skeletal metastases should be encouraged.

### 5.4 Telehealth for older patients with cancer

During COVID‐19 outbreak, health‐care systems have had to adjust the way they triage, evaluate, and care for patients using methods that do not rely on in‐person services in order to limit hospital visits, to avoid oversaturation of medical facilities, to reduce exposure to potentially ill persons and to preserve personal protective equipment.

Telehealth services may help provide necessary care to patients while minimizing the transmission risk of SARS‐CoV‐2. While telehealth technology and its use are not new, widespread adoption among health‐care professionals and patients has been relatively slow.^76^, ^77^

Even if some increased interest in use of telehealth services has been seen in the last years,^78^, ^79^, ^80^ recent policy changes during the COVID‐19 pandemic have reduced barriers to telehealth access and fostered its use as a way to deliver acute, chronic, primary, and specialty care.^81^

Telemedicine has been shown to improve patient outcomes in non‐oncological settings especially by lowering readmission rates, which is of pivotal importance for older patients during the pandemic.^82^

Patients’ and caregivers’ satisfaction for telemedicine lacks evidence, since no clear definition nor measurement are available, yet, a recent systematic review in a non‐oncological setting found there were high levels of satisfaction across several assessed domains relating to telemedicine.^80^ Some early data in the general cancer population point at a fair general acceptance of telemedicine, as well as of telehealth multidisciplinary geriatric oncology clinics.^83^

Given this background, telehealth services for older cancer patients should be particularly encouraged during the COVID‐19 pandemic for delivering geriatric assessment,^84^ for management of treatment‐related toxicity, for follow‐up, as well as for pre‐habilitation programs.^63^

## 6 CONCLUSION

Managing care for older cancer patients poses a major challenge during the COVID‐19 pandemic. If in the last 20 years life expectancy has increased enough to make it necessary to think about how to customize treatments for older people with cancer, today's scenario poses numerous questions, ethical doubts, and difficulties in making choices. The balance between undertreatment and overtreatment is made tougher by the incumbent risk of potentially fatal infections which are very frequent in older subjects, especially older patients with cancer. Access to Intensive Care Units (ICUs) raises ethical questions, as well as response to treatments and medical concerns. All indications currently available in international guidelines need a constant reassessment, which takes into account patients’ characteristics and the context in which they are located, from the probability of infection in a low‐risk city to the possibility of having granted access to the ICU.

Even more in these times it becomes essential not to use solely chronological age as choice criteria but to evaluate case by case, basing the therapeutic choice on a correct and thorough assessment of all the variables.

In conclusion, when approaching older cancer patients in the midst of the COVID‐19 pandemic it is imperative to enhance multidimensional assessment to guide decisions, to create a safe environment, to implement telemedicine to improve care delivery while accommodating physical distancing, always considering the ethical impact of professional guidelines for treatment prioritization.

## CONFLICT OF INTEREST

Antonella Brunello: Consultancy/Advisory: Eisai – Eli Lilly – Roche; Travel grants: Pharmamar – Ipsen. Vittorina Zagonel: Consultancy/Advisory Bristol‐Myers Squibb, Merck; Speakers’ bureau: Bristol‐Myers Squibb, Merck, Astellas Pharma, Servier, AstraZeneca, Lilly; Travel grants: Bayer, Roche, Servier. Antonella Galiano, Silvia Finotto, Giuseppe Colloca, Lodovico Balducci, and Silvio Monfardini do not declare any conflict of interest.

## AUTHOR CONTRIBUTIONS

All authors contributed to conception and design, drafting, and critically revising the manuscript, and give final approval to the version to be published.

## Data Availability

Data sharing not applicable to this article as no data sets were generated or analyzed during the current study.
